# *Calonectria* in the age of genes and genomes: Towards understanding an important but relatively unknown group of pathogens

**DOI:** 10.1111/mpp.13209

**Published:** 2022-03-25

**Authors:** JieQiong Li, Michael J. Wingfield, Irene Barnes, ShuaiFei Chen

**Affiliations:** ^1^ Department of Biochemistry, Genetics and Microbiology, Forestry and Agricultural Biotechnology Institute University of Pretoria Pretoria South Africa; ^2^ Research Institute of Fast‐growing Trees/China Eucalypt Research Centre, Chinese Academy of Forestry Zhanjiang China

**Keywords:** *Cylindrocladium*, genomics, global distribution, mating type, plant pathogen

## Abstract

The genus *Calonectria* includes many aggressive plant pathogens causing diseases on various agricultural crops as well as forestry and ornamental tree species. Some species have been accidentally introduced into new environments via international trade of putatively asymptomatic plant germplasm or contaminated soil, resulting in significant economic losses. This review provides an overview of the taxonomy, population biology, and pathology of *Calonectria* species, specifically emerging from contemporary studies that have relied on DNA‐based technologies. The growing importance of genomics in future research is highlighted. A life cycle is proposed for *Calonectria* species, aimed at improving our ability to manage diseases caused by these pathogens.

## Graphical abstract

The taxonomy, population biology, pathology, and genomics of *Calonectria*, an important but relatively unknown group of pathogens of agricultural crops as well as forestry and ornamental trees, are reviewed.

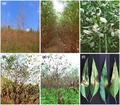

## 1 INTRODUCTION

*Calonectria* (*Ca*.) is an ascomycete fungus that resides in one of 47 genera of the Nectriaceae (Lombard et al., 2015b; Rossman et al., 1999). The genus was originally erected in 1867 (De Notaris, 1867) based on the sexual morph of *Calonectria daldiniana* (a synonym of *Ca. pyrochroa*; Rossman, 1979) isolated from leaves of *Magnolia grandiflora* in Italy (Crous, 2002; Vitale et al., 2013). Later, Morgan (1892) described the asexual morph that has branched conidiophores, cylindrical conidia, and stipe extensions with characteristic terminal vesicles (Crous & Wingfield, 1994) in the genus *Cylindrocladium* (*Cy*.).

For many years, species of *Calonectria* were best known by their asexual *Cylindrocladium* morphs. This was largely due to the fact that the asexual state is the one most commonly encountered on diseased plants. The dual nomenclature system applied for pleomorphic fungi meant that both asexual and sexual names were used in the literature, often interchangeably and sometimes inconsistently, resulting in considerable confusion (Hawksworth et al., 2011; Wingfield et al., 2012). For example, *Cy. parasiticum* is the asexual name for *Ca. ilicicola* (Crous et al., 1993b; Lechat et al., 2010), while the sexual name for *Cy*. *ilicicola* is *Ca. lauri* (Lechat et al., 2010). This confusing situation continued until the acceptance of the “one fungus, one name” system (Hawksworth et al., 2011; McNeill et al., 2012; Wingfield et al., 2012). All *Cylindrocladium* species were then transferred to *Calonectria*, which is the oldest available name, and this was irrespective of whether a sexual state was known or not.

*Calonectria* presently accommodates 126 species. Of these, *Ca. curvata*, *Ca. hederae* and *Ca. pyrochroa* are species for which there are no ex‐type cultures or DNA sequences available (Crous, 2002; Lombard et al., 2010b; Marin‐Felix et al., 2017; Rossman, 1979). All other species have been defined based on DNA barcode gene comparisons and morphological features (Crous et al., 2018, 2019; Liu et al., 2020; Wang et al., 2019). These species reside in two main phylogenetic clades known as the prolate and the sphaeronaviculate groups, names that refer to the distinct shapes of the vesicles emerging from their conidiogenous apparatuses (Liu et al., 2020; Lombard et al., 2010b; Marin‐Felix et al., 2017).

**FIGURE 1:**
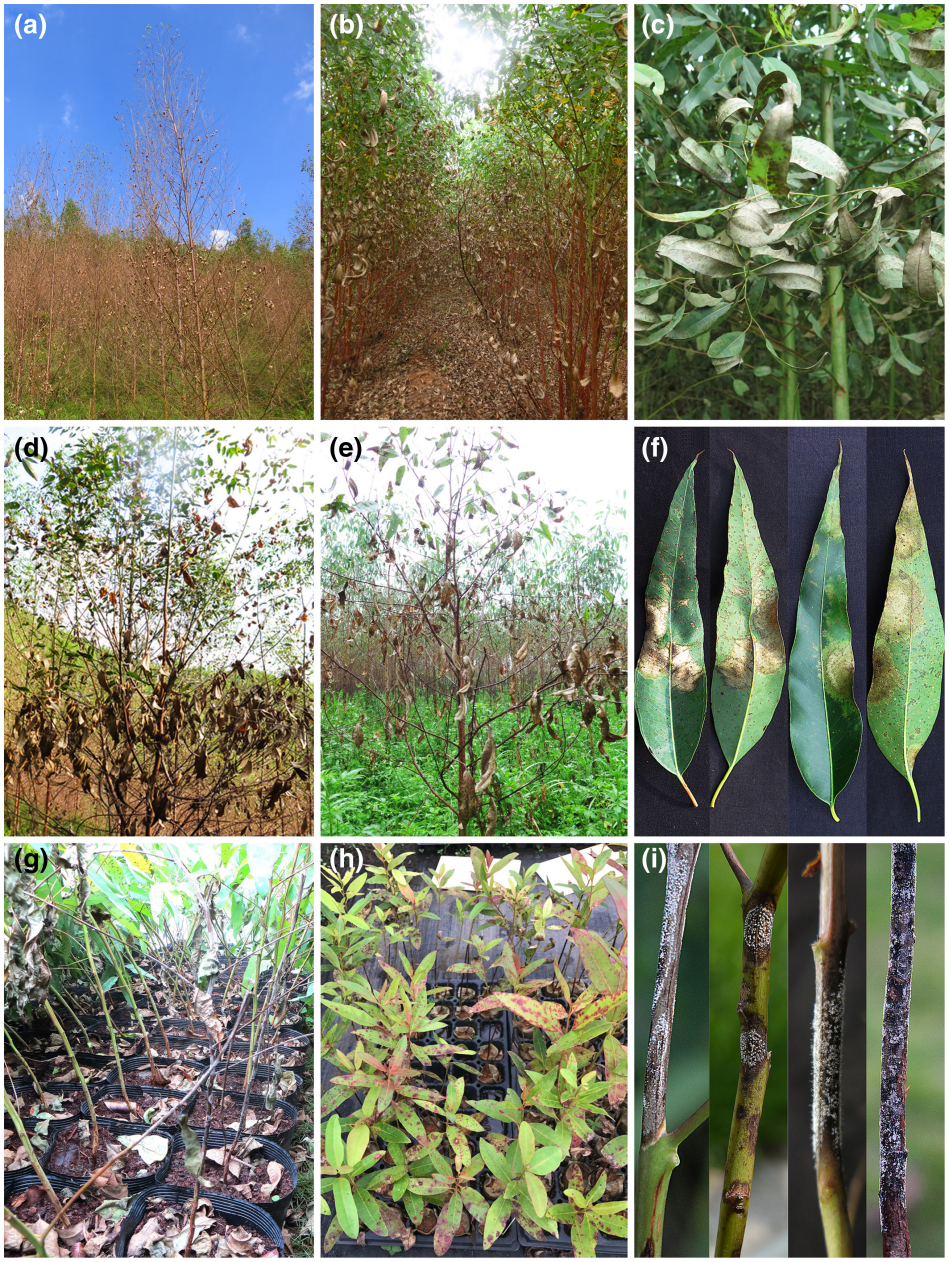
Disease symptoms on *Eucalyptus* spp., including hybrids of *E. urophylla* with *E. grandis* (a, b, and d), *E. tereticornis* (c and f), and *E. pellita* (e) caused by species of *Calonectria*. (a)–(f) Disease symptoms in *Eucalyptus* plantations. (a) Tree death after infection. (b) Defoliation associated with leaf and shoot blight. (c) Early stage of leaf blight after infection. (d) and (e) Leaf blight. (f) Typical blight symptoms on leaf front and back. (g)–(i) Disease symptoms on *Eucalyptus* nursery plants. (g) Stems of infected *E. urophylla* ×* E*. *tereticornis* hybrid seedlings. (h) Leaves of infected *E*. *urophylla* seedlings. (i) Stems of *E. urophylla* ×* E*. *grandis* hybrid seedlings blackened due to rot and covered with profuse white sporulation of the pathogen

When they were first discovered, species of *Calonectria* were considered as saprophytes (Graves, 1915). The first disease associated with these fungi was crown canker (Massey, 1917), caused by *Ca. cylindrospora* (*Cy*. *scoparium* = *Ca*. *morganii*) (Alfenas et al., 2015; Crous, 2002; Lombard et al., 2015b) on *Rosa* spp. Subsequently, *Calonectria* species were recognized as agents of important diseases on many agricultural, forestry, and horticultural plants (Crous, 2002; Liu et al., 2020; Lombard et al., 2010c). Approximately 335 plant species residing in about 100 plant families are hosts of *Calonectria* species (Crous, 2002; Lombard et al., 2010c). About 66 of the 126 *Calonectria* species are known to cause disease on a wide variety of plant hosts (Crous et al., 2018, 2019; Liu et al., 2020; Wang et al., 2019; Wu & Chen, 2021). Disease symptoms (Figure 1) resulting from these pathogens include seedling and cutting rot, leaf and shoot blight, leaf spot, defoliation, root rot, and stem canker (Crous, 2002; Liu et al., 2021b; Lombard et al., 2010c; Wu & Chen, 2021).

Most studies on *Calonectria* have focused on the taxonomy, phylogeny, and pathogenicity of species (Lombard et al., 2010c, 2016; Marin‐Felix et al., 2017). However, with the development of molecular biological techniques in recent years, our understanding of *Calonectria* has changed dramatically. The aim of this review is to illustrate how data relating to genes and genomes have significantly changed and influenced our understanding of the taxonomy and population genetic diversity in *Calonectria*. We also consider how a rapidly growing resource of genome data has already impacted, and will increasingly influence, our understanding of the pathogenicity in these important fungi.

## 2 TAXONOMY OF *CALONECTRIA*

**FIGURE 2:**
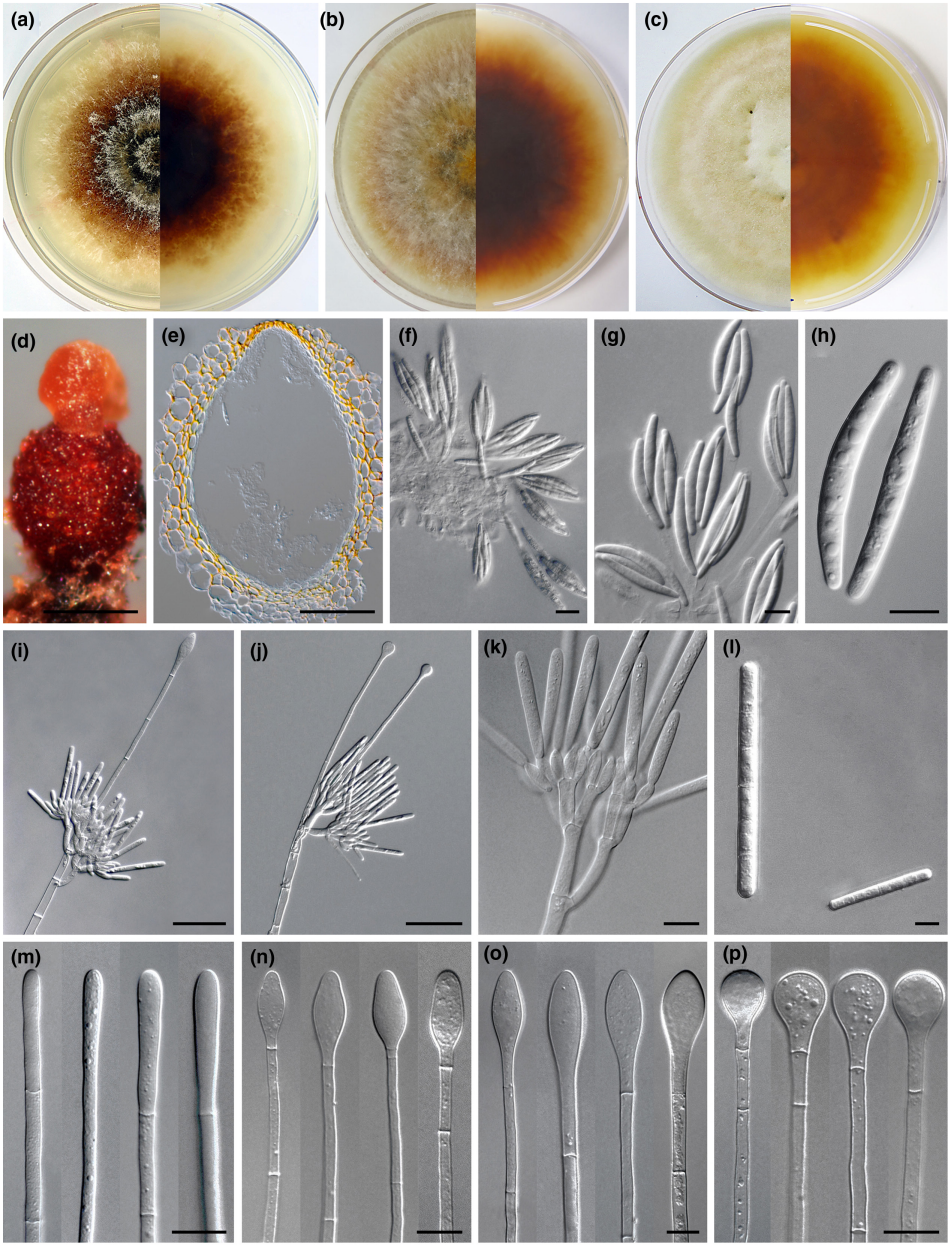
Cultural and morphological characteristics of *Calonectria* spp. (a)–(c) Three different colony morphologies of *Calonectria* species on malt extract agar after growth at 25°C in the dark for 7 days: (a) *Ca*. *honghensis* CERC 5572, (b) *Ca*. *kyotensis* CERC 7126, and (c) *Ca*. *yunnanensis* CERC 5339. (d)–(h) Sexual morphs: (d) orange perithecium of *Ca. kyotensis* CERC 7126, (e) vertical section through a perithecium of *Ca*. *aconidialis* CERC 9937, (f) asci of *Ca*. *aconidialis* CERC 9937 with eight ascospores, (g) asci of *Ca*. *honghensis* CERC 5572 with four ascospores, and (h) ascospores of *Ca*. *honghensis* CERC 5572 with three septa. (i)–(p) Asexual morphs: (i) macroconidiophore with an ellipsoidal vesicle in *Ca. cerciana* CMW 37972, (j) macroconidiophore with a sphaeropedunculate vesicle in *Ca. heveicola* CMW 49928, (k) conidiogenous apparatus of *Ca. heveicola* CMW 49928 with conidiophore branches and doliiform to reniform phialides, (l) macro‐ and microconidia of *Ca*. *reteaudii* CSF 23967, (m) clavate vesicles of *Ca*. *honghensis* CERC 5572, (n) obpyriform vesicles of *Ca. brevistipitata* CBS 110837, (o) ellipsoidal vesicle of *Ca. cerciana* CMW 37972, and (p) sphaeropedunculate vesicles of *Ca. cochinchinensis* CBS 143569. Scale bars: (d) 200 μm, (e) 100 μm, (f) 20 μm, (g), (h) and (k)–(p) 10 μm, (i) and (j) 50 μm

For many years subsequent to the first description of the genus, species of *Calonectria* were identified based on their morphological differences (Figure 2). Asexual morphs treated in *Cylindrocladium* were recognized as providing more distinguishing characters than sexual morphs (Crous, 2002; Liu et al., 2020; Lombard et al., 2010b, 2016; Rossman, 1979). This especially concerned the shapes and diameters of the vesicles as well as the septation and dimensions of the conidia (Crous, 2002; Lombard et al., 2010b, 2016; Schoch et al., 2000a). However, this approach was complicated by the fact that morphological variation between some species was, at best, subtle, leading to incorrect identification of species and the fact that cryptic species were commonly overlooked (Alfenas et al., 2015; Crous, 2002; Liu et al., 2020; Lombard et al., 2010a, 2010b, 2016; Schoch et al., 1999, 2000b).

The biological species concept (BSC) is used to define a taxon as a group of organisms that can successfully interbreed and produce fertile offspring (Ereshefsky, 2007; Sokal & Crovello, 1970); the same biological species can be recognized by sexual compatibility within a species and the reproductive isolation between different species. This approach was first applied to the taxonomy of *Calonectria* by Crous et al. (1993a) and it was extensively used for this purpose in later studies (Crous, 2002; Liu et al., 2020; Lombard et al., 2010a; Schoch et al., 1999, 2000b).

Most species of *Calonectria* are self‐sterile and have a biallelic heterothallic mating system (Crous, 2002; Crous et al., 1998; Li et al., 2020). Consequently, some cryptic species have been recognized based on their mating compatibility (Lombard et al., 2010a; Schoch et al., 1999). However, limitations have arisen in applying the biological species concept to the taxonomy of *Calonectria*. For example, inducing fruiting bodies in the laboratory for *Calonectria* spp. is time consuming, requiring up to 2 months (Crous, 2002; Lombard et al., 2010a; Schubert et al., 1989). Sexual recombination is also a complex process related to the genetic properties of strains and is strongly influenced by the environment (Goodenough & Heitman, 2014). Thus, the conditions used to conduct mating studies in the laboratory are not uniformly conducive to achieve reliable results. Moreover, some species lose their ability to recombine and produce fertile progeny under laboratory conditions (Crous, 2002; Lombard et al., 2010a), implying that mating tests fail in culture.

### 2.1 Gene and gene regions applied in the taxonomy of *Calonectria*

The emergence of DNA sequencing technology has contributed greatly to our capacity to recognize cryptic species and to identify unknown species. The phylogenetic species concept emphasizes nucleotide divergence between monophyletic lineages, which uses phylogenetic analysis of sequence variation to define species (Cai et al., 2011; Taylor et al., 2000). The Fungal Barcode of Life project has further highlighted the importance of a universal DNA barcode that could be used for the identification of all fungal species (Seifert, 2009).

In 1997, the internal transcribed spacer regions and intervening 5.8S nrRNA gene (ITS) was applied as the barcoding gene to distinguish between species of *Calonectria* (Jeng et al., 1997). However, a low number of informative characters was available in the DNA sequence of this genetic region (Crous et al., 2000; Schoch et al., 1999). Later, Crous et al. (2000) found that the β‐tubulin (*tub2*) gene provided better resolution than the ITS for the identification of these fungi.

Taylor et al. (2000) established and recommended genealogical concordance phylogenetic species recognition, a high‐resolution approach in which multiple gene genealogies could be used to separate isolates into genetically distinct species. Thus, between the years 2000 and 2010, gene genealogy analyses of calmodulin (*cmdA*), histone H3 (*his3*), translation elongation factor 1‐α (*tef1*), and *tub2* sequences were frequently used to identify *Calonectria* spp. (Crous et al., 2004, 2006; Lombard et al., 2009, 2010d). Subsequently, Lombard et al. (2010b) amplified seven different gene regions for 66 *Calonectria* species to screen for the best gene regions to delimit species in this genus. These gene regions included actin (*act*), *cmdA*, *his3*, ITS, *tef1*, *tub2*, and 28S nuclear ribosomal large subunit (LSU). The results showed that the *cmdA* gene region provided the best resolution to distinguish between closely related species of *Calonectria*. Furthermore, the *his3*, *tef1*, and *tub2* regions alone provided useful resolution (Lombard et al., 2010b).

Subsequent to the study of Lombard et al. (2010b), the application of DNA sequences for multiple gene regions was widely used in taxonomic studies on *Calonectria* (Alfenas et al., 2015; Chen et al., 2011; Gehesquière et al., 2016; Li et al., 2017; Lombard et al., 2016; Marin‐Felix et al., 2017; Pham et al., 2019). Most recently, Liu et al. (2020) reconsidered the species boundaries based on 169 *Calonectria* species using eight gene regions, including *act*, *cmdA*, *his3*, ITS, LSU, DNA‐directed RNA polymerase II subunit (*rpb2*), *tef1*, and *tub2*. A combination of six gene regions (*tef1*, *tub2*, *cmdA*, *his3*, *rpb2*, and *act*) was found to provide the best resolution and a stable basis for the identification of *Calonectria* species. Liu et al. (2020) proposed that these six gene regions be routinely used as effective barcodes for species in the genus.

Approximately 60 novel *Calonectria* spp. have been identified based on the multigene phylogenetic species concept approach in the last 10 years (Crous et al., 2018, 2019; Liu et al., 2020; Lombard et al., 2010b; Wang et al., 2019). The multigene phylogenetic species concept has thus had profound implications for the taxonomy of species in this genus. However, the phenomenon of conflicting gene trees has emerged as a common problem (Rokas & Carroll, 2006). Debates surrounding the multigene phylogenetic species concept continue and there remain many open questions. These include how many unlinked genes are necessary to reveal cryptic species (Balasundaram et al., 2015; Jeewon & Hyde, 2016; Taylor et al., 2000), how much sequence divergence should there be within a fragment of a gene to define a species (Jeewon & Hyde, 2016; Lukhtanov, 2019), what bootstrap value should be accepted to support new lineages (Hillis & Bull, 1993; Lukhtanov, 2019), and whether all genes selected suitably reflect the evolutionary history of the genus (Hillis & Bull, 1993; Lukhtanov, 2019).

### 2.2 Genomes applied to the taxonomy of fungi

The recent and rapidly growing availability of genome sequences makes phylogenomic, as opposed to phylogenetic, analyses possible for all organisms (Delsuc et al., 2005; Dornburg et al., 2017; Robbertse et al., 2006). It increases the phylogenetic signal and drives a robust resolution by joining the sequences from large numbers of gene regions (Delsuc et al., 2005; Rintoul et al., 2012). This method has increasingly been successfully applied to the taxonomy of fungi (Ascunce et al., 2017; Dornburg et al., 2017; Kanzi et al., 2020), including important pathogens such as species of *Fusarium* (Fourie et al., 2013; Geiser et al., 2021; Villani et al., 2015), rust fungi (Aime et al., 2017), and oomycetes (McCarthy & Fitzpatrick, 2017). However, there have been no phylogenomic analyses for species of *Calonectria*, although it is inevitable that this situation will change in the near future.

## 3 POPULATION GENETICS AND THE DISEASE CYCLE

Even though *Calonectria* has been known since the mid‐19th century, very little work has been done to determine the population structure and diversity of populations for even the most important species. The earliest such study was conducted by Schoch et al. (2001), and employed mating tests and DNA sequence comparisons for the *tub2* gene region to investigate genetic variation among *Ca. pauciramosa* populations from Italy, South Africa, and the United States. Later, Wright et al. (2006) developed microsatellite markers for *Ca. ilicicola* and subsequently for *Ca. pauciramosa* (Wright et al., 2007) using a random amplified microsatellite approach. Furthermore, Wright et al. (2010) used these microsatellite markers to determine the genetic diversity of *Ca. ilicicola* populations associated with peanuts (*Arachis hypogaea*) in the United States.

Subsequent to the study of Wright et al. (2010), there was little work on the populations of *Calonectria* spp. for approximately 10 years. In last 2 years, four such studies have been published (Freitas et al., 2019; LeBlanc et al., 2019; Li et al., 2021; Malapi‐Wight et al., 2019). Three of these used whole‐genome data to design genetic markers to reveal the global population structure of *Ca. henricotiae*, *Ca. pauciramosa*, and *Ca. pseudonaviculata* (LeBlanc et al., 2019; Li et al., 2021; Malapi‐Wight et al., 2019). The remaining study used inter‐simple sequence repeat markers to determine the genetic diversity of *Ca*. *pteridis* (Freitas et al., 2019). This provided the first evidence that genome data is emerging as an important tool to promote an enhanced understanding of the population biology of *Calonectria* species that cause important plant diseases.

**FIGURE 3:**
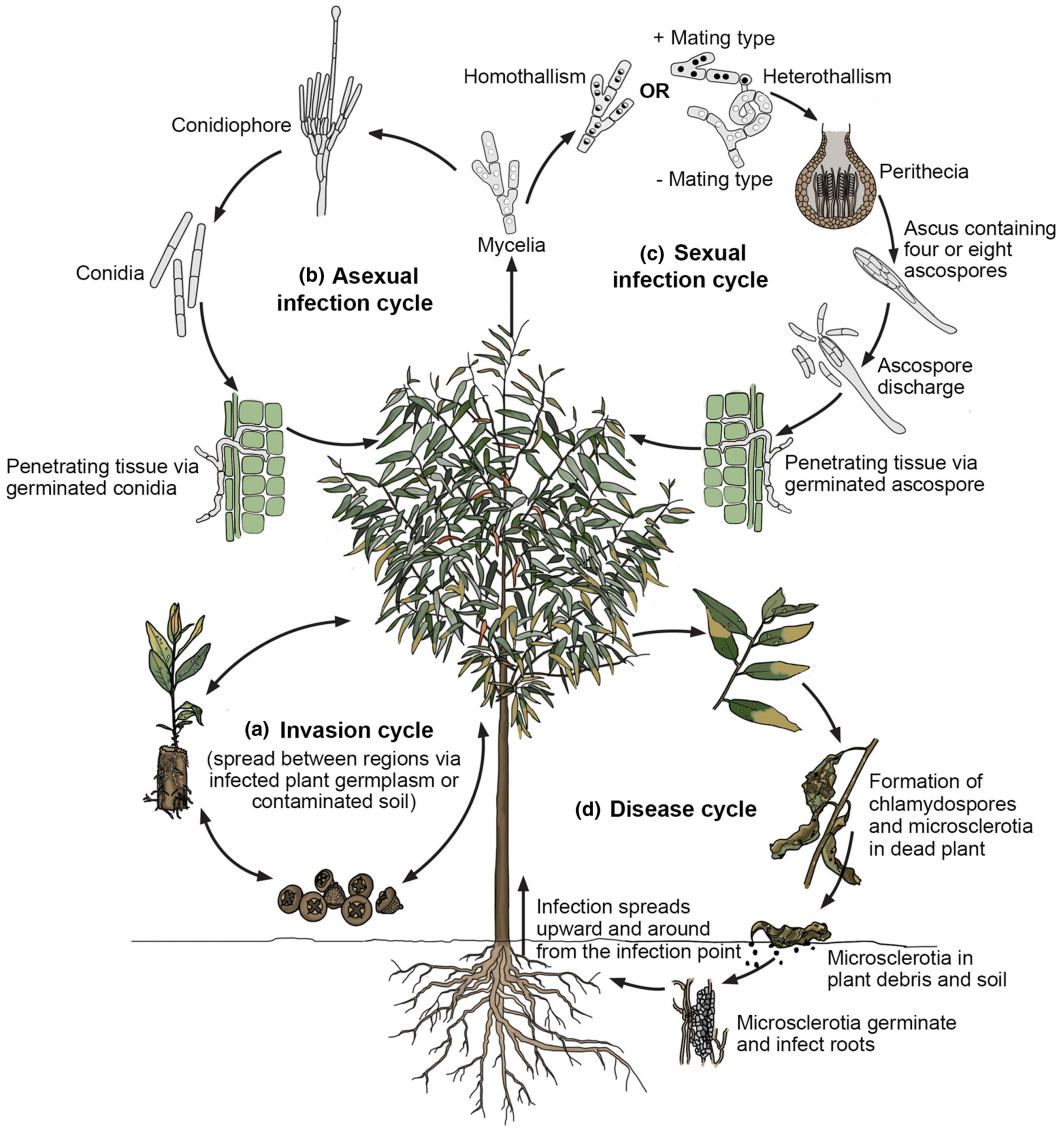
Putative life cycle of *Calonectria* species. (a) *Calonectria* pathogens spread between regions via infected plant germplasm or contaminated soil. (b) Once *Calonectria* is introduced, the propagules germinate to form mycelium on the surface of infected plants under suitable environmental conditions. Mycelium can rapidly initiate the asexual cycle by forming a large number of conidiophores in a short period of time. (c) Under unfavourable conditions, the pathogen can enter the sexual cycle by the union of individuals of opposite mating type (heterothallism) or self‐fertilization (homothallism). After mate recognition, cell–cell fusion, and diploid zygote formation, gametes are generated via meiosis and ploidy changes via mitosis (Ni et al., 2011; Wilson et al., 2019). The haploid ascospores are formed and dispersed by wind or rain splash to penetrate healthy plant tissue. (d) Infections usually begin from the base of a tree or seedling and lead to various disease symptoms (Chen et al., 2011; Rodas et al., 2005). The long‐term survival structures are microsclerotia that can be found in plant debris and soil (Crous, 2002; Phipps et al., 1976). When conditions are suitable for growth to occur, microsclerotia germinate to infect roots and the disease cycle is repeated

All previous studies concerning the population biology of pathogenic *Calonectria* species have revealed an intriguing and important fact. This is that a single dominant genotype of various species is widely distributed in many countries. These species include *Ca. henricotiae* in Europe (LeBlanc et al., 2019), *Ca. ilicicola* in the United States (Wright et al., 2010), *Ca. pauciramosa* in Africa, Asia, Europe, North America, and Oceania (Li et al., 2021), *Ca. pseudonaviculata* in Asia, Europe, New Zealand, and North America (LeBlanc et al., 2019), and *Ca. pteridis* in Brazil (Freitas et al., 2019). These clonal *Calonectria* populations in many different countries clearly implicate a global movement of infected plant germplasm or soil (Figure 3a) (Burgess & Wingfield, 2017; Wingfield et al., 2001). This also reflects trends in agriculture, forestry, and the ornamental plant trade that contribute deeply to the global distribution of pathogens (Roy, 2016; Santini et al., 2018; Wingfield et al., 2001), including *Calonectria* species, via international trade and travel.

Once a pathogenic *Calonectria* species has been accidentally introduced into a new environment (Figure 3), it is able to rapidly colonize and adapt by producing a large number of asexual propagules (conidia, chlamydospores, and microsclerotia) in a short period of time (Ashu & Xu, 2015; Crous et al., 1991; Vitale et al., 2013). This is consistent with the fact that conidia of the asexual stage of *Calonectria* species are often observed on diseased plant tissues. Conidia are the propagules that enable direct penetration of healthy plant tissue (Crous, 2002; West, 2014). Chlamydospores usually occur in clusters and form microsclerotia, which are specialized long‐term survival structures that can be found in soil and host tissue, enabling the fungus to resist harsh environments (Pérez‐Sierra et al., 2007; Phipps et al., 1976). When conditions are suitable for growth to occur, microsclerotia germinate to produce hyphae and conidia that then infect plants (Avenot et al., 2017; Dart et al., 2015).

The asexual propagules of *Calonectria* species are able to disperse over short distances aided by rain splash or in irrigation water, in wind currents, via insect vectors, and farming tools, and thus to cause local disease epidemics (Crous, 2002; Crous et al., 1991; Vitale et al., 2013). In addition, once individuals of opposite mating type in a heterothallic *Calonectria* species are introduced into the same area and come into contact, the pathogen is able to produce new and potentially more aggressive genotypes via sexual recombination, which can be dispersed over longer distances (Crow, 1994; Heitman et al., 2013; Lumley et al., 2015). This then increases their adaptability to the environment and their ability to break down resistance genes in plant hosts, leading to disease outbreaks (Ashu & Xu, 2015; McDonald & Linde, 2002).

*Calonectria* spp. can have one of two modes of sexual reproduction (Alfieri et al., 1982; Schubert et al., 1989). Thus, some species are heterothallic whereas others are homothallic (Crous, 2002; Li et al., 2020; Lombard et al., 2010c). In heterothallic species, individual isolates, derived from a single spore, will have either one of the two mating type idiomorphs. These individuals are self‐sterile and require a compatible partner to mate and produce fertile sexual structures (Kronstad & Staben, 1997; Ni et al., 2011). In contrast, homothallic species are self‐fertile, where an individual derived from a single spore possesses both mating type idiomorphs and can, therefore, complete the sexual cycle on its own (Kronstad & Staben, 1997; Wilson et al., 2015).

The mating type distribution in heterothallic pathogens provides important information making it possible to predict whether sexual reproduction might be occurring, and thus to understand the population dynamics and evolutionary potential of pathogens (Bakhshi et al., 2011; Glass & Kuldau, 1992; Turgeon, 1998). Sexual reproduction in *Calonectria* is based on a bipolar mating system, controlled by mating type (*MAT*) genes that are found at a single *MAT* locus (*MAT1*) with two nonallelic forms, the *MAT1‐1* and *MAT1‐2* idiomorphs (Turgeon & Yoder, 2000; Yoder et al., 1986). Based on genome sequences, heterothallic species residing in *Calonectria* have commonly been found to harbour the *MAT1‐1‐1*, *MAT1‐1‐2*, and *MAT1‐1‐3* genes in the *MAT1‐1* idiomorph, and the *MAT1‐2‐1* and *MAT1‐2‐13* genes in the *MAT1‐2* idiomorph (Li et al., 2020; Malapi‐Wight et al., 2019; Wilson et al., 2021).

As molecular techniques have been developed, mating type markers have emerged to provide rapid and accurate tools to monitor the mating type distribution in populations of important *Calonectria* species. Using mating type markers, Malapi‐Wight et al. (2019) found, in a collection of the important boxwood blight pathogens from four continents, that all *Ca. henricotiae* isolates were of the MAT1‐1 mating type, whereas all isolates of *Ca. pseudonaviculata* were MAT1‐2. Likewise, in a global population diversity study of the aggressive pathogen *Ca. pauciramosa*, Li et al. (2021) showed that the MAT1‐2 mating type was present in isolates from every continent sampled. Furthermore, they found that only the MAT1‐1 mating type was present in isolates from Africa and South America. These results provided evidence of accidental introductions into new areas and suggested the need for improved quarantine measures to prevent introductions of strains of the opposite mating type.

## 4 AN IMPROVED UNDERSTANDING OF PATHOGENICITY

*Calonectria* species are important aggressive pathogens of many different plants and are widely distributed in different regions of the world. For example, *Ca*. *pseudonaviculata* is regarded as a considerable threat to boxwood (*Buxus* spp.) in Europe and North America (Daughtrey, 2019; Gehesquière et al., 2013, 2016), where these plants are commonly propagated as ornamentals. Other species, including *Ca. pauciramosa*, have caused serious diseases on numerous woody, herbaceous, and ornamental plants worldwide, especially in *Eucalyptus* plantations of South Africa and South America (Crous, 2002; Crous et al., 1991; Li et al., 2021; Lombard et al., 2010a; Schoch et al., 2001). *Ca. pteridis* is one of the most important foliar pathogens of *Eucalyptus* spp. in Brazil (Alfenas et al., 2015, 2016; Ferreira et al., 1995; Freitas et al., 2019; Graça et al., 2009). Similarly, *Ca. pseudoreteaudii* is the causal agent of Calonectria leaf blight on *Eucalyptus* in plantations of China and Southeast Asia (Crous et al., 2012; Li et al., 2017; Liu et al., 2021b; Lombard et al., 2010d, 2015a; Wang & Chen, 2020; Ye et al., 2017, 2018). Thus, understanding the biology and particularly the mechanisms underlying pathogenicity in *Calonectria* species is emerging as an important topic for future research, particularly as this relates to disease prevention and control.

There have been few studies on *Calonectria* species regarding the mechanisms underpinning pathogenicity that have used genome sequencing technologies. The only such investigations have been those of Ye et al. (2017, 2018) and Santos et al. (2020), who analysed the mechanisms of pathogenicity in *Ca. pseudoreteaudii* on *Eucalyptus*. They suggested that the establishment of Calonectria leaf blight is associated with toxin and cell‐wall‐degrading enzymes (Santos et al., 2020; Ye et al., 2017, 2018). Clearly, there are many opportunities to better understand the biology of *Calonectria* species and their modes of pathogenicity. In this regard, future studies to consider these factors will depend on the availability of whole‐genome sequences for *Calonectria* species. These are rapidly emerging and it is realistic to expect that genomes of most species of *Calonectria* spp. will become available for study in the near future.

An important first step towards understanding the molecular basis of fungal pathogenicity in plants is the availability of a reliable and meaningful inoculation protocol. For *Calonectria* species, conidial suspensions or mycelial plugs placed on whole plants or detached leaves are commonly used to test for pathogenicity (Alfieri et al., 1972; El‐Gholl et al., 1993; Graça et al., 2009; Guo et al., 2016; Richardson et al., 2020; Wang & Chen, 2020). These techniques are beset by a number of challenges. For example, various species of *Calonectria* fail to sporulate in culture, making inoculation using spores impossible. In these cases, it is common to use agar plugs overgrown with mycelium or mycelial fragments in inoculation tests. It remains unclear whether the latter approach mirrors the natural situation and consequently studies considering genetic responses to infection could be compromised. Thus, future studies on the molecular mechanisms underpinning the pathogenicity of *Calonectria* species will require refined techniques to inoculate plants.

## 5 CONCLUSIONS

This review has highlighted the manner in which molecular biological techniques have changed, but also influenced our understanding of the taxonomy, population diversity, and pathogenicity of *Calonectria* species. The emergence and ready availability of genome sequencing has played an important part in this development. To date (2022), the genomes of 13 *Calonectria* species have been sequenced, and these are available on the public genome databases at NCBI (Table 1, https://www.ncbi.nlm.nih.gov/genome/). These genomes have been used to define the population genetics and evolution of *Calonectria* species based on their microsatellite sites and mating type loci (LeBlanc et al., 2019; Li et al., 2020, 2021; Malapi‐Wight et al., 2019), and they have highlighted factors related to pathogenicity in *Ca. pseudoreteaudii* on *Eucalyptus* (Santos et al., 2020; Ye et al., 2017, 2018). We anticipate that the genomes of all *Calonectria* species available in culture will become available for study in the relatively near future. This will substantially promote our understanding of these fungi.

**TABLE 1:** Details of all *Calonectria* species for which genomes have been sequenced

No.	Species^a^	Isolate number	Genome	Assembly size (Mb)	GC (%)	Scaffold/contig number	Coverage	N50 (bp)	Host	References
1	*Ca. aciculate*	**CBS 142883** ^b^ **; CMW 47645; CERC 5342**	VTGE01000000	61.60	47.72	221	40	675,696	*Eucalyptus urophylla* × *E*. *grandis* leaf	Li et al. (2017); Liu et al. (2019)
2	*Ca. crousiana*	**CBS 127198; CMW 27249**	VTGD01000000	58.10	48.72	358	114	419,924	*E. grandis*	Chen et al. (2011); Liu et al. (2019)
3	*Ca. fujianensis*	**CBS 127201; CMW 27257**	VTGC01000000	61.50	46.81	194	68	695,013	*E. grandis*	Chen et al. (2011); Liu et al. (2019)
4	*Ca. henricotiae*	**CBS 138102; CB045**	PGWR00000000	53.70	48.90	9527	70	15,400	*Buxus sempervirens*	Crouch et al. (2017); Gehesquière et al. (2016); Malapi‐Wight et al. (2019)
		CB077^c^	PGSE00000000	47.50	49.89	5907	30	17,113	*B. sempervirens*	Crouch et al. (2017); Gehesquière et al. (2016)
		NL009^c^	PGSF01000000	49.10	49.08	10,129	34	12,040	*B. sempervirens*	Crouch et al. (2017); Gehesquière et al. (2016)
		NL017^c^	PHMY00000000	43.30	49.78	28,983	32	1787	*B. sempervirens*	Crouch et al. (2017)
5	*Ca. honghensis*	**CBS 142885; CMW 47669; CERC 5572**	VTGB01000000	61.70	47.36	141	76	1,034,491	Soil (*Eucalyptus* plantation)	Li et al. (2017); Liu et al. (2019)
6	*Ca. hongkongensis*	CMW 47271; CERC 3570	JAACJA000000000	61.70	48.99	76	737	1,665,378	Soil (*Eucalyptus* plantation)	Li et al. (2017); Li et al. (2020)
7	*Ca. ilicicola*	F018	PRJNA672291	68.97	46.58	16	92	6,053,737	*Glycine max*	Liu et al. (2021a)
		FJLY41	JACVOE010000000	70.22	46.20	294	73	641,047	*Arachis hypogaea*	Gai et al. (2020); Liu et al. (2021a)
		GDBL01	JACVOJ010000001	68.63	46.60	320	72	478,317	*Glycine max*	Gai et al. (2020); Liu et al. (2021a)
		GDBL02	JACVOI010000001	69.57	46.70	338	72	555,473	*A. hypogaea*	Gai et al. (2020); Liu et al. (2021a)
		GDBL60	JACVOH010000001	68.75	46.50	325	74	510,771	*G. max*	Gai et al. (2020); Liu et al. (2021a)
		GDMZ12	JACVOG010000001	69.88	46.50	343	72	520,651	*A. hypogaea*	Gai et al. (2020); Liu et al. (2021a)
		GDZQ186	JADDLS010000001	69.74	46.50	350	72	438,287	*A. hypogaea*	Gai et al. (2020); Liu et al. (2021a)
		JXLN31	JACVOF010000001	69.86	46.50	475	73	432,030	*A. hypogaea*	Gai et al. (2020); Liu et al. (2021a)
		ZJHZ01	JACVOD010000001	70.36	46.50	301	73	697,969	*G. max*	Gai et al. (2020); Liu et al. (2021a)
8	*Ca. leucothoës*	**CBS 109166; CMW 30977; CPC 2385**	NAJI00000000	63.10	49.50	3373	124	253,300	*Leucothoe axillaris*	Malapi‐Wight et al. (2019); Lombard et al. (2010b, 2010d)
9	*Ca. naviculata*	**CBS 101121; CMW 30974**	NAGG00000000	65.70	50.50	5768	84	55,957	Leaf litter	Crouch et al. (2017); Malapi‐Wight et al. (2019); Lombard et al. (2010b)
10	*Ca. pauciramosa*	**CBS 138824; CMW 5683; CPC 971**	JAACIZ000000000	62.40	49.29	83	1015	3,148,270	*E. grandis*	Li et al. (2020); Lombard et al. (2010a, 2010b)
		CMW 7592	JAACIY000000000	62.30	49.34	104	895	1,368,225	*E. grandis*	Li et al. (2020); Lombard et al. (2010a, 2010b)
11	*Ca. pseudonaviculata*	**CBS 114417** ^c^ **; CMW 23672; CPC 10926**	RQSK00000000	47.30	50.06	5830	19	16,962	*B. sempervirens*	Crouch et al. (2017); Lombard et al. (2010b)
		CBS 139394	JYJY00000000	51.40	47.86	1340	70	121,364	*Sarcococca hookeriana*	Malapi‐Wight et al. (2016b)
		CBS 139707	PGGA00000000	55.00	46.40	27	83	3,534,400	*B. sempervirens*	Malapi‐Wight et al. (2016a, 2016b, 2019)
		CB002^c^	RQSK00000000	49.40	48.52	7679	81	17,153	*B. sempervirens*	Crouch et al. (2017); Gehesquière et al. (2016); LeBlanc et al. (2019)
		CT13^c^	PGWW00000000	47.80	49.79	5104	49	20,929	*B. sempervirens*	Crouch et al. (2017)
		ICMP 14368^c^	PHNA00000000	30.80	49.73	26,060	15	1371	*B. sempervirens*	Crouch et al. (2017)
		NC‐BB1^c^	PHMZ00000000	39.80	49.69	21,917	16	2592	*B. sempervirens*	Crouch et al. (2017)
		ODA1^c^	PHNB00000000	45.20	50.13	16,359	16	4587	*B. sempervirens*	Crouch et al. (2017); Gehesquière et al. (2016)
12	*Ca. pseudoreteaudii*	YA51	MOCD00000000	63.70	48.30	507	213	1,316,000	*Eucalyptus* sp.	Ye et al. (2018)
13	*Ca. pseudoturangicola*	**CBS 142890; CMW 47496; CERC 7126**	VTGA01000000	62.10	47.67	155	79	875,460	Soil	Li et al. (2017); Liu et al. (2019)

^a^ CBS, Westerdijk Fungal Biodiversity Institute, Utrecht, Netherlands; CERC, China Eucalypt Research Centre, Chinese Academy of Forestry, Zhanjiang, GuangDong Province, China; CMW, culture collection of the Forestry and Agricultural Biotechnology Institute (FABI), University of Pretoria, Pretoria, South Africa; CPC, Pedro Crous working collection housed at CBS; CB, CT, F, FJLY, GDBL, GDMZ, GDZQ, ICMP, JXLN, NC‐BB1, ODA1, YA, ZJHZ, Personal working culture collection numbers.

^b^ Isolates represented by ex‐type cultures are indicated in bold.

^c^ The genome assembly quality was evaluated using the abyss‐fac function of ABySS (Jackman et al., 2017) in this review.

[Correction added on 23 April 2022, after first online publication: the 'Country’ column in Table 1 has been deleted in this version.]

While species of *Calonectria* have been collected and studied where they are associated with diseases of crop plants, there has been a strong bias towards particular environments such as their presence in *Eucalyptus* plantations (Liu et al., 2020; Lombard et al., 2010b). This is linked to the fact that they have emerged as important constraints to *Eucalyptus* plantation forestry, particularly where these trees are propagated as non‐natives (Alfenas et al., 2015; Freitas et al., 2019; Li et al., 2017, 2021; Liu et al., 2021b; Schoch et al., 2001; Wang & Chen, 2020; Wu & Chen, 2021; Ye et al., 2017, 2018). Where they have emerged on *Eucalyptus*, most species have probably originated as part of the natural soil environment. This suggests a significant gap in our knowledge and a need to sample soils in natural forests and other ecosystems. Sampling crop environments other than those linked to forestry and also the many parts of the world where these fungi have not been considered should be a priority in the future.

While genome sequencing has already impacted substantially on *Calonectria* research, next‐generation sequencing and metagenomic studies have not been undertaken with a focus on these pathogens. As with other fungi (Stewart et al., 2018; Tremblay et al., 2018; Vaz et al., 2017), such studies will make it possible to more deeply interrogate questions relating to the presence of *Calonectria* species in the soil and other environments where they might not easily be detected using culture‐dependent approaches. They will also improve quarantine protocols and reduce accidental introductions of pathogenic *Calonectria* species into new environments.

## CONFLICT OF INTEREST

The authors declare no conflict of interest.

## Data Availability

Data sharing is not applicable to this article as no new data were created or analysed.

## References

[mpp13209-bib-0001] Aime, M.C. , McTaggart, A.R. , Mondo, S.J. & Duplessis, S. (2017) Phylogenetics and phylogenomics of rust fungi. Advances in Genetics, 100, 267–307. 29153402 10.1016/bs.adgen.2017.09.011

[mpp13209-bib-0002] Alfenas, R.F. , Freitas, R.G. , Pereira, O.L. , Coutinho, M.M. , Zarpelon, T.G. , Candido, T.S. et al. (2016) Screening of *Corymbia* and *Eucalyptus* species for resistance to *Calonectria pteridis* leaf blight. Forest Pathology, 46, 76–81.

[mpp13209-bib-0003] Alfenas, R.F. , Lombard, L. , Pereira, O.L. , Alfenas, A.C. & Crous, P.W. (2015) Diversity and potential impact of *Calonectria* species in *Eucalyptus* plantations in Brazil. Studies in Mycology, 80, 89–130. 26955192 10.1016/j.simyco.2014.11.002 PMC4779794

[mpp13209-bib-0004] Alfieri, S.A. , El‐Gholl, N.E. & Schoulties, C.L. (1982) Homothallism in *Calonectria ilicicola* . Mycologia, 74, 513–514.

[mpp13209-bib-0005] Alfieri, S.A., Jr. , Linderman, R.G. , Morrison, R.H. & Sobers, E.K. (1972) Comparative pathogenicity of *Calonectria theae* and *Cylindrocladium scoparium* . Phytopathology, 62, 647–650.

[mpp13209-bib-0006] Ascunce, M.S. , Huguet‐Tapia, J.C. , Ortiz‐Urquiza, A. , Keyhani, N.O. , Braun, E.L. & Goss, E.M. (2017) Phylogenomic analysis supports multiple instances of polyphyly in the oomycete peronosporalean lineage. Molecular Phylogenetics and Evolution, 114, 199–211. 28645766 10.1016/j.ympev.2017.06.013

[mpp13209-bib-0007] Ashu, E.E. & Xu, J. (2015) The roles of sexual and asexual reproduction in the origin and dissemination of strains causing fungal infectious disease outbreaks. Infection, Genetics and Evolution, 36, 199–209. 10.1016/j.meegid.2015.09.019 26394109

[mpp13209-bib-0008] Avenot, H.F. , King, C. , Edwards, T.P. , Baudoin, A. & Hong, C.X. (2017) Effects of inoculum dose, temperature, cultivar, and interrupted leaf wetness period on infection of boxwood by *Calonectria pseudonaviculata* . Plant Disease, 101, 866–873. 30682940 10.1094/PDIS-05-16-0742-RE

[mpp13209-bib-0009] Bakhshi, M. , Arzanlou, M. & Babai‐Ahari, A. (2011) Uneven distribution of mating type alleles in Iranian populations of *Cercospora beticola*, the causal agent of *Cercospora* leaf spot disease of sugar beet. Phytopathologia Mediterranea, 50, 101–109.

[mpp13209-bib-0010] Balasundaram, S.V. , Engh, I.B. , Skrede, I. & Kauserud, H. (2015) How many DNA markers are needed to reveal cryptic fungal species? Fungal Biology, 119, 940–945. 26399188 10.1016/j.funbio.2015.07.006

[mpp13209-bib-0011] Burgess, T.I. & Wingfield, M.J. (2017) Pathogens on the move: a 100‐year global experiment with planted eucalypts. BioScience, 67, 14–25.

[mpp13209-bib-0012] Cai, L. , Giraud, T. , Zhang, N. , Begerow, D. , Cai, G. & Shivas, R.G. (2011) The evolution of species concepts and species recognition criteria in plant pathogenic fungi. Fungal Diversity, 50, 121–133.

[mpp13209-bib-0013] Chen, S.F. , Lombard, L. , Roux, J. , Xie, Y.J. , Wingfield, M.J. & Zhou, X.D. (2011) Novel species of *Calonectria* associated with *Eucalyptus* leaf blight in Southeast China. Persoonia, 26, 1–12. 22025800 10.3767/003158511X555236 PMC3160799

[mpp13209-bib-0014] Crouch, J. , Malapi‐Wight, M. , Rivera, Y. , Salgado‐Salazar, C. & Veltri, D. (2017) Genome datasets for *Calonectria henricotiae* and *C. pseudonaviculata* causing boxwood blight disease and related species. Ag Data Commons. 10.15482/USDA.ADC/1410184

[mpp13209-bib-0015] Crous, P.W. (2002) Taxonomy and pathology of *Cylindrocladium* (*Calonectria*) and allied genera. St Paul, MN: American Phytopathological Society Press.

[mpp13209-bib-0016] Crous, P.W. , Alfenas, A.C. & Junghans, T.G. (1998) Variability within *Calonectria ovata* and its anamorph *Cylindrocladium ovatum* from Brazil. Sydowia, 50, 1–13.

[mpp13209-bib-0017] Crous, P.W. , Carnegie, A.J. , Wingfield, M.J. , Sharma, R. , Mughini, G. , Noordeloos, M.E. et al. (2019) Fungal Planet description sheets: 868–950. Persoonia, 42, 291–473. 31551622 10.3767/persoonia.2019.42.11 PMC6712538

[mpp13209-bib-0018] Crous, P.W. , Groenewald, J.Z. , Risède, J.M. , Simoneau, P. & Hyde, K.D. (2006) *Calonectria* species and their *Cylindrocladium* anamorphs: species with clavate vesicles. Studies in Mycology, 55, 213–226. 18490981 10.3114/sim.55.1.213 PMC2104717

[mpp13209-bib-0019] Crous, P.W. , Groenewald, J.Z. , Risède, J.M. , Simoneau, P. & Hywel‐Jones, N.L. (2004) *Calonectria* species and their *Cylindrocladium* anamorphs: species with sphaeropedunculate vesicles. Studies in Mycology, 50, 415–430. 10.3114/sim.55.1.213 PMC2104717 18490981

[mpp13209-bib-0020] Crous, P.W. , Kang, J.C. , Schoch, C.L. & Mchau, G.R. (2000) Phylogenetic relationships of *Cylindrocladium pseudogracile* and *Cylindrocladium rumohrae* with morphologically similar taxa, based on morphology and DNA sequences of internal transcribed spacers and β‐tubulin. Canadian Journal of Botany, 77, 1813–1820.

[mpp13209-bib-0021] Crous, P.W. , Luangsa‐ard, J.J. , Wingfield, M.J. , Carnegie, A.J. , Hernández‐Restrepo, M. , Lombard, L. et al. (2018) Fungal Planet description sheets: 785–867. Persoonia, 41, 238–417. 30728607 10.3767/persoonia.2018.41.12 PMC6344811

[mpp13209-bib-0022] Crous, P.W. , Phillips, A.J.L. & Wingfield, M.J. (1991) The genera *Cylindrocladium* and *Cylindrocladiella* in South Africa, with special reference to forest nurseries. South African Forestry Journal, 157, 69–85.

[mpp13209-bib-0023] Crous, P.W. , Shivas, R.G. , Wingfield, M.J. , Summerell, B.A. , Rossman, A.Y. , Alves, J.L. et al. (2012) Fungal Planet description sheets: 128–153. Persoonia, 29, 146–201. 23606771 10.3767/003158512X661589 PMC3589791

[mpp13209-bib-0024] Crous, P.W. & Wingfield, M.J. (1994) A monograph of *Cylindrocladium*, including anamorphs of *Calonectria* . Mycotaxon, 51, 341–435.

[mpp13209-bib-0025] Crous, P.W. , Wingfield, M.J. & Alfenas, A.C. (1993a) Additions to *Calonectria* . Mycotaxon, 46, 217–234.

[mpp13209-bib-0026] Crous, P.W. , Wingfield, M.J. & Alfenas, A.C. (1993b) *Cylindrocladium parasiticum* sp. nov., a new name for *C. crotalariae* . Mycological Research, 97, 889–896.

[mpp13209-bib-0027] Crow, J.F. (1994) Advantages of sexual reproduction. Developmental Genetics, 15, 205–213. 8062455 10.1002/dvg.1020150303

[mpp13209-bib-0028] Dart, N. , Hong, C. , Craig, C.A. , Fry, J.T. & Hu, X. (2015) Soil inoculum production, survival, and infectivity of the boxwood blight pathogen, *Calonectria pseudonaviculata* . Plant Disease, 99, 1689–1694. 30699514 10.1094/PDIS-12-14-1245-RE

[mpp13209-bib-0029] Daughtrey, M.L. (2019) Boxwood blight: threat to ornamentals. Annual Review of Phytopathology, 57, 189–209. 10.1146/annurev-phyto-082718-100156 31283434

[mpp13209-bib-0030] De Notaris, G. (1867) Nuove reclute per la pirenomicetologia italica. Commentario della Società Crittogamologica Italiana, 2, 477–492.

[mpp13209-bib-0031] Delsuc, F. , Brinkmann, H. & Philippe, H. (2005) Phylogenomics and the reconstruction of the tree of life. Nature Reviews Genetics, 6, 361–375. 10.1038/nrg1603 15861208

[mpp13209-bib-0032] Dornburg, A. , Townsend, J.P. & Wang, Z. (2017) Maximizing power in phylogenetics and phylogenomics: a perspective illuminated by fungal big data. Advances in Genetics, 100, 1–47. 29153398 10.1016/bs.adgen.2017.09.007

[mpp13209-bib-0033] El‐Gholl, N.E. , Alfenas, A.C. , Crous, P.W. & Schubert, T.S. (1993) Description and pathogenicity of *Cylindrocladium ovatum* sp. nov. Canadian Journal of Botany, 71, 466–470.

[mpp13209-bib-0034] Ereshefsky, M. (2007) Species, taxonomy, and systematics. In: Mauthen, M. & Stephens, C. (Eds.) Philosophy of biology. Amsterdam: North‐Holland, pp. 403–427.

[mpp13209-bib-0035] Ferreira, F.A. , Alfenas, A.C. , Moreira, A.M. & Demuner, N.L. (1995) Pteridis spot eucalypt leaf disease in tropical areas of Brazil. Brazil Phytopathology, 20, 107–110.

[mpp13209-bib-0036] Fourie, G. , van der Merwe, N.A. , Wingfield, B.D. , Bogale, M. , Tudzynski, B. , Wingfield, M.J. et al. (2013) Evidence for inter‐specific recombination among the mitochondrial genomes of *Fusarium* species in the *Gibberella fujikuroi* complex. BMC Genomics, 14, 605. 24010864 10.1186/1471-2164-14-605 PMC3847072

[mpp13209-bib-0037] Freitas, R.G. , Alfenas, R.F. , Guimarães, L.M.S. , Badel, J.L. & Alfenas, A.C. (2019) Genetic diversity and aggressiveness of *Calonectria pteridis* in *Eucalyptus* spp. Plant Pathology, 68, 869–877.

[mpp13209-bib-0038] Gai, Y.P. , Liu, B. , Li, L. , Ma, H.J. , Peng, X.J. , Pan, R.Q. et al. (2020) The Genome sequences of *Calonectria ilicicola* (anamorph *Cylindrocladium parasiticum*) causing Cylindrocladium black rot of peanut and red crown rot of soybean [Data set]. Zenodo. 10.5281/zenodo.4147239

[mpp13209-bib-0039] Gehesquière, B. , Crouch, J.A. , Marra, R.E. , Van Poucke, K. , Rys, F. , Maes, M. et al. (2016) Characterization and taxonomic reassessment of the box blight pathogen *Calonectria pseudonaviculata*, introducing *Calonectria henricotiae* sp. nov. Plant Pathology, 65, 37–52.

[mpp13209-bib-0040] Gehesquière, B. , D'Haeyer, S. , Pham, K.T.K. , Van Kuik, A.J. , Maes, M. , Höfte, M. et al. (2013) qPCR assays for the detection of *Cylindrocladium buxicola* in plant, water, and air samples. Plant Disease, 97, 1082–1090. 30722484 10.1094/PDIS-10-12-0964-RE

[mpp13209-bib-0041] Geiser, D.M. , Al‐Hatmi, A.M. , Aoki, T. , Arie, T. , Balmas, V. , Barnes, I. , et al. (2021) Phylogenomic analysis of a 55.1 kb 19‐gene dataset resolves a monophyletic *Fusarium* that includes the *Fusarium solani* species complex. Phytopathology, 111, 1064–1079. 33200960 10.1094/PHYTO-08-20-0330-LE

[mpp13209-bib-0042] Glass, N.L. & Kuldau, G.A. (1992) Mating type and vegetative incompatibility in filamentous ascomycetes. Annual Review of Phytopathology, 30, 201–224. 10.1146/annurev.py.30.090192.001221 18643772

[mpp13209-bib-0043] Goodenough, U. & Heitman, J. (2014) Origins of eukaryotic sexual reproduction. Cold Spring Harbor Perspectives in Biology, 6, a016154. 24591519 10.1101/cshperspect.a016154 PMC3949356

[mpp13209-bib-0044] Graça, R.N. , Alfenas, A.C. , Maffia, L.A. , Titon, M. , Alfenas, R.F. , Lau, D. et al. (2009) Factors influencing infection of eucalypts by *Cylindrocladium pteridis* . Plant Pathology, 58, 971–981.

[mpp13209-bib-0045] Graves, A.H. (1915) Root rot of coniferous seedlings. Phytopathology, 5, 213–217.

[mpp13209-bib-0046] Guo, Y. , Olsen, R.T. , Kramer, M. & Pooler, M. (2016) Use of mycelium and detached leaves in bioassays for assessing resistance to boxwood blight. Plant Disease, 100, 1622–1626. 30686223 10.1094/PDIS-01-16-0016-RE

[mpp13209-bib-0047] Hawksworth, D.L. , Crous, P.W. , Redhead, S.A. , Reynolds, D.R. , Samson, R.A. , Seifert, K.A. et al. (2011) The Amsterdam declaration on fungal nomenclature. IMA Fungus, 2, 105–111. 22679594 10.5598/imafungus.2011.02.01.14 PMC3317370

[mpp13209-bib-0048] Heitman, J. , Sun, S. & James, T.Y. (2013) Evolution of fungal sexual reproduction. Mycologia, 105, 1–27. 23099518 10.3852/12-253

[mpp13209-bib-0049] Hillis, D.M. & Bull, J.J. (1993) An empirical test of bootstrapping as a method for assessing confidence in phylogenetic analysis. Systematic Biology, 42, 182–192.

[mpp13209-bib-0050] Jackman, S.D. , Vandervalk, B.P. , Mohamadi, H. , Chu, J. , Yeo, S. , Hammond, S.A. et al. (2017) ABySS 2.0: resource‐efficient assembly of large genomes using a Bloom filter. Genome Research, 27, 768–777. 28232478 10.1101/gr.214346.116 PMC5411771

[mpp13209-bib-0051] Jeewon, R. & Hyde, K.D. (2016) Establishing species boundaries and new taxa among fungi: recommendations to resolve taxonomic ambiguities. Mycosphere, 7, 1669–1677.

[mpp13209-bib-0052] Jeng, R.S. , Dumas, M. , Liu, F.H. , Wang, C.L. & Hubbes, M. (1997) DNA analysis of *Cylindrocladium floridanum* isolates from selected forest nurseries. Mycological Research, 101, 285–291.

[mpp13209-bib-0053] Kanzi, A.M. , Trollip, C. , Wingfield, M.J. , Barnes, I. , Van der Nest, M.A. & Wingfield, B.D. (2020) Phylogenomic incongruence in *Ceratocystis*: a clue to speciation? BMC Genomics, 21, 362. 32408859 10.1186/s12864-020-6772-0 PMC7222570

[mpp13209-bib-0054] Kronstad, J.W. & Staben, C. (1997) Mating type in filamentous fungi. Annual Review of Genetics, 31, 245–276. 10.1146/annurev.genet.31.1.245 9442896

[mpp13209-bib-0055] LeBlanc, N. , Gehesquière, B. , Salgado‐Salazar, C. , Heungens, K. & Crouch, J.A. (2019) Limited genetic diversity across pathogen populations responsible for the global emergence of boxwood blight identified using SSRs. Plant Pathology, 68, 861–868.

[mpp13209-bib-0056] Lechat, C. , Crous, P.W. & Groenewald, J.Z. (2010) The enigma of *Calonectria* species occurring on leaves of *Ilex aquifolium* in Europe. IMA Fungus, 1, 101–108. 22679568 10.5598/imafungus.2010.01.02.01 PMC3348779

[mpp13209-bib-0057] Li, J.Q. , Barnes, I. , Liu, F.F. , Wingfield, M. & Chen, S.F. (2021) Global genetic diversity and mating type distribution of *Calonectria pauciramosa*: an important wide host‐range plant pathogen. Plant Disease, 105, 1648–1656. 33200973 10.1094/PDIS-05-20-1050-RE

[mpp13209-bib-0058] Li, J.Q. , Wingfield, B.D. , Wingfield, M.J. , Barnes, I. , Fourie, A. , Crous, P.W. et al. (2020) Mating genes in *Calonectria* and evidence for a heterothallic ancestral state. Persoonia, 45, 163–176. 34456375 10.3767/persoonia.2020.45.06 PMC8375350

[mpp13209-bib-0059] Li, J.Q. , Wingfield, M.J. , Liu, Q.L. , Barnes, I. , Roux, J. , Lombard, L. et al. (2017) *Calonectria* species isolated from *Eucalyptus* plantations and nurseries in South China. IMA Fungus, 8, 259–286. 29242775 10.5598/imafungus.2017.08.02.04 PMC5729712

[mpp13209-bib-0060] Liu, F.F. , Chen, S.F. , Ferreira, M.A. , Chang, R.L. , Sayari, M. , Kanzi, A.M. et al. (2019) Draft genome sequences of five *Calonectria* species from *Eucalyptus* plantations in China, *Celoporthe dispersa*, *Sporothrix phasma* and *Alectoria sarmentosa* . IMA Fungus, 10, 22. 32647626 10.1186/s43008-019-0023-5 PMC7325655

[mpp13209-bib-0061] Liu, H.H. , Wang, J. , Wu, P.H. , Lu, M.Y.J. , Li, J.Y. , Shen, Y.M. et al. (2021a) The whole genome sequence resource of *Calonectria ilicicola*, the casual pathogen of soybean red crown rot. Molecular Plant‐Microbe Interactions, 34, 848–851. 33683143 10.1094/MPMI-11-20-0315-A

[mpp13209-bib-0062] Liu, L.L. , Wu, W.X. & Chen, S.F. (2021b) Species diversity and distribution characteristics of *Calonectria* in five soil layers in a *Eucalyptus* plantation. Journal of Fungi, 7, 857. 34682278 10.3390/jof7100857 PMC8541508

[mpp13209-bib-0063] Liu, Q.L. , Li, J.Q. , Wingfield, M.J. , Duong, T.A. , Wingfield, B.D. , Crous, P.W. et al. (2020) Reconsideration of species boundaries and proposed DNA barcodes for *Calonectria* . Studies in Mycology, 97, 100106. 34322181 10.1016/j.simyco.2020.08.001 PMC8295567

[mpp13209-bib-0064] Lombard, L. , Chen, S.F. , Mou, X. , Zhou, X.D. , Crous, P.W. & Wingfield, M.J. (2015a) New species, hyper‐diversity and potential importance of *Calonectria* spp. from *Eucalyptus* in South China. Studies in Mycology, 80, 151–188. 26955194 10.1016/j.simyco.2014.11.003 PMC4779793

[mpp13209-bib-0065] Lombard, L. , Crous, P.W. , Wingfield, B.D. & Wingfield, M.J. (2010a) Multigene phylogeny and mating tests reveal three cryptic species related to *Calonectria pauciramosa* . Studies in Mycology, 66, 15–30. 20806004 10.3114/sim.2010.66.02 PMC2886098

[mpp13209-bib-0066] Lombard, L. , Crous, P.W. , Wingfield, B.D. & Wingfield, M.J. (2010b) Phylogeny and systematics of the genus *Calonectria* . Studies in Mycology, 66, 31–69. 20806005 10.3114/sim.2010.66.03 PMC2886099

[mpp13209-bib-0067] Lombard, L. , Crous, P.W. , Wingfield, B.D. & Wingfield, M.J. (2010c) Species concepts in *Calonectria* (*Cylindrocladium*). Studies in Mycology, 66, 1–13. 20806003 10.3114/sim.2010.66.01 PMC2886097

[mpp13209-bib-0068] Lombard, L. , Rodas, C.A. , Crous, P.W. , Wingfield, B.D. & Wingfield, M.J. (2009) *Calonectria* (*Cylindrocladium*) species associated with dying *Pinus* cuttings. Persoonia, 23, 41–47. 20198160 10.3767/003158509X471052 PMC2802723

[mpp13209-bib-0069] Lombard, L. , Van der Merwe, N.A. , Groenewald, J.Z. & Crous, P.W. (2015b) Generic concepts in Nectriaceae. Studies in Mycology, 80, 189–245. 26955195 10.1016/j.simyco.2014.12.002 PMC4779799

[mpp13209-bib-0070] Lombard, L. , Wingfield, M.J. , Alfenas, A.C. & Crous, P.W. (2016) The forgotten *Calonectria* collection: pouring old wine into new bags. Studies in Mycology, 85, 159–198. 28082759 10.1016/j.simyco.2016.11.004 PMC5220189

[mpp13209-bib-0071] Lombard, L. , Zhou, X.D. , Crous, P.W. , Wingfield, B.D. & Wingfield, M.J. (2010d) *Calonectria* species associated with cutting rot of *Eucalyptus* . Persoonia, 24, 1–11. 20664755 10.3767/003158510X486568 PMC2890164

[mpp13209-bib-0072] Lukhtanov, V.A. (2019) Species delimitation and analysis of cryptic species diversity in the XXI century. Entomological Review, 99, 463–472.

[mpp13209-bib-0073] Lumley, A.J. , Michalczyk, Ł. , Kitson, J.J.N. , Spurgin, L.G. , Morrison, C.A. , Godwin, J.L. et al. (2015) Sexual selection protects against extinction. Nature, 522, 470–473. 25985178 10.1038/nature14419

[mpp13209-bib-0074] Malapi‐Wight, M. , Demers, J.E. , Veltri, D. , Marra, R.E. & Crouch, J.A. (2016a) LAMP detection assays for boxwood blight pathogens: a comparative genomics approach. Scientific Reports, 6, 26140. 27199028 10.1038/srep26140 PMC4873745

[mpp13209-bib-0075] Malapi‐Wight, M. , Salgado‐Salazar, C. , Demers, J.E. , Clement, D.L. , Rane, K.K. & Crouch, J.A. (2016b) Sarcococca blight: use of whole‐genome sequencing for fungal plant disease diagnosis. Plant Disease, 100, 1093–1100. 30682271 10.1094/PDIS-10-15-1159-RE

[mpp13209-bib-0076] Malapi‐Wight, M. , Veltri, D. , Gehesquière, B. , Heungens, K. , Rivera, Y. , Salgado‐Salazar, C. et al. (2019) Global distribution of mating types shows limited opportunities for mating across populations of fungi causing boxwood blight disease. Fungal Genetics and Biology, 131, 103246. 31254611 10.1016/j.fgb.2019.103246

[mpp13209-bib-0077] Marin‐Felix, Y. , Groenewald, J.Z. , Cai, L. , Chen, Q. , Marincowitz, S. , Barnes, I. et al. (2017) Genera of phytopathogenic fungi: GOPHY 1. Studies in Mycology, 86, 99–216. 28663602 10.1016/j.simyco.2017.04.002 PMC5486355

[mpp13209-bib-0078] Massey, L.M. (1917) The crown canker disease of rose. Phytopathology, 7, 408–417.

[mpp13209-bib-0079] McCarthy, C.G.P. & Fitzpatrick, D.A. (2017) Phylogenomic reconstruction of the oomycete phylogeny derived from 37 genomes. mSphere, 2, e00095‐17. 28435885 10.1128/mSphere.00095-17 PMC5390094

[mpp13209-bib-0080] McDonald, B.A. & Linde, C. (2002) Pathogen population genetics, evolutionary potential, and durable resistance. Annual Review of Phytopathology, 40, 349–379. 10.1146/annurev.phyto.40.120501.101443 12147764

[mpp13209-bib-0081] McNeill, J. , Barrie, F.R. , Buck, W.R. , Demoulin, V. , Greuter, W. , Hawksworth, D.L. et al. (2012) International Code of Nomenclature for algae, fungi and plants (Melbourne Code). Königstein: Koeltz Scientific Books.

[mpp13209-bib-0082] Morgan, A.P. (1892) Two new genera of hyphomycetes. Botanical Gazette, 17, 190–192.

[mpp13209-bib-0083] Ni, M. , Feretzaki, M. , Sun, S. , Wang, X. & Heitman, J. (2011) Sex in fungi. Annual Review of Genetics, 45, 405–430. 10.1146/annurev-genet-110410-132536 PMC3310392 21942368

[mpp13209-bib-0084] Pérez‐Sierra, A. , Alvarez, L.A. , Leon, M. , Abad‐Campos, P. , Armengol, J. & Garcia‐Jimenez, J. (2007) First report of leaf spot, blight, and stem lesions caused by *Cylindrocladium pauciramosum* on callistemon in Spain. Plant Disease, 91, 1057. 10.1094/PDIS-91-8-1057C 30780461

[mpp13209-bib-0085] Pham, N.Q. , Barnes, I. , Chen, S.F. , Liu, F.F. , Dang, Q.N. , Pham, T.Q. et al. (2019) Ten new species of *Calonectria* from Indonesia and Vietnam. Mycologia, 111, 78–102. 30657437 10.1080/00275514.2018.1522179

[mpp13209-bib-0086] Phipps, P.M. , Beute, M.K. & Barker, K.R. (1976) An elutriation method for quantitative isolation of *Cylindrocladium crotalariae* microsclerotia from peanut field soil. Phytopathology, 66, 1255–1259.

[mpp13209-bib-0087] Richardson, P.A. , Daughtrey, M. & Hong, C. (2020) Indications of susceptibility to *Calonectria pseudonaviculata* in some common groundcovers and boxwood companion plants. Plant Disease, 104, 1127–1132. 32040391 10.1094/PDIS-08-19-1582-RE

[mpp13209-bib-0088] Rintoul, T.L. , Eggertson, Q.A. & Lévesque, C.A. (2012) Multigene phylogenetic analyses to delimit new species in fungal plant pathogens. In: Bolton, M.D. & Thomma, B.P.H.J. (Eds.) Plant fungal pathogens. New York, NY: Humana Press, pp. 549–569. 10.1007/978-1-61779-501-5_34 22183677

[mpp13209-bib-0089] Robbertse, B. , Reeves, J.B. , Schoch, C.L. & Spatafora, J.W. (2006) A phylogenomic analysis of the Ascomycota. Fungal Genetics and Biology, 43, 715–725. 16781175 10.1016/j.fgb.2006.05.001

[mpp13209-bib-0090] Rodas, C.A. , Lombard, L. , Gryzenhoinf, M. , Slippers, B. & Wingfield, M.J. (2005) *Cylindrocladium* blight of *Eucalyptus grandis* in Colombia. Australasian Plant Pathology, 34, 143–149.

[mpp13209-bib-0091] Rokas, A. & Carroll, S.B. (2006) Bushes in the tree of life. PLoS Biology, 4, e352. 17105342 10.1371/journal.pbio.0040352 PMC1637082

[mpp13209-bib-0092] Rossman, A.Y. (1979) *Calonectria* and its type species, *C. daldiniana*, a later synonym of *C. pyrochroa* . Mycotaxon, 8, 321–328.

[mpp13209-bib-0093] Rossman, A.Y. , Samuels, G.J. , Rogerson, C.T. & Lowen, R. (1999) Genera of Bionectriaceae, Hypocreaceae and Nectriaceae (Hypocreales, Ascomycetes). Studies in Mycology, 42, 1–248.

[mpp13209-bib-0094] Roy, H. (2016) Control wildlife pathogens too. Nature, 530, 281. 10.1038/530281d 26887485

[mpp13209-bib-0095] Santini, A. , Liebhold, A. , Migliorini, D. & Woodward, S. (2018) Tracing the role of human civilization in the globalization of plant pathogens. The ISME Journal, 12, 647–652. 29330537 10.1038/s41396-017-0013-9 PMC5864165

[mpp13209-bib-0096] Santos, S.A. , Vidigal, P.M.P. , Thrimawithana, A. , Betancourth, B.M.L. , Guimarães, L.M.S. , Templeton, M.D. et al. (2020) Comparative genomic and transcriptomic analyses reveal different pathogenicity‐related genes among three eucalyptus fungal pathogens. Fungal Genetics and Biology, 137, 103332. 31926322 10.1016/j.fgb.2019.103332

[mpp13209-bib-0097] Schoch, C.L. , Crous, P.W. , Polizzi, G. & Koike, S.T. (2001) Female fertility and single nucleotide polymorphism comparisons in *Cylindrocladium pauciramosum* . Plant Disease, 85, 941–946. 30823106 10.1094/PDIS.2001.85.9.941

[mpp13209-bib-0098] Schoch, C.L. , Crous, P.W. , Wingfield, B.D. & Wingfield, M.J. (1999) The *Cylindrocladium candelabrum* species complex includes four distinct mating populations. Mycologia, 91, 286–298.

[mpp13209-bib-0099] Schoch, C.L. , Crous, P.W. , Wingfield, M.J. & Wingfield, B.D. (2000a) Phylogeny of *Calonectria* and selected hypocrealean genera with cylindrical macroconidia. Studies in Mycology, 45, 45–62.

[mpp13209-bib-0100] Schoch, C.L. , Crous, P.W. , Witthuhn, R.C. , Cronwright, G. , El‐Gholl, N.E. & Wingfield, B.D. (2000b) Recombination in *Calonectria morganii* and phylogeny with other heterothallic small‐spored *Calonectria* species. Mycologia, 92, 665–673.

[mpp13209-bib-0101] Schubert, T.S. , El‐Gholl, N.E. , Alfieri, S.A., Jr. & Schoulties, C.L. (1989) *Calonectria avesiculata* sp. nov. Canadian Journal of Botany, 67, 2414–2419.

[mpp13209-bib-0102] Seifert, K.A. (2009) Progress towards DNA barcoding of fungi. Molecular Ecology Resources, 9, 83–89. 10.1111/j.1755-0998.2009.02635.x 21564968

[mpp13209-bib-0103] Sokal, R.R. & Crovello, T.J. (1970) The biological species concept: a critical evaluation. The American Naturalist, 104, 127–153.

[mpp13209-bib-0104] Stewart, J.E. , Kim, M.S. & Klopfenstein, N.B. (2018) Molecular genetic approaches toward understanding forest‐associated fungi and their interactive roles within forest ecosystems. Current Forestry Reports, 4, 72–84.

[mpp13209-bib-0105] Taylor, J.W. , Jacobson, D.J. , Kroken, S. , Kasuga, T. , Geiser, D.M. , Hibbett, D.S. et al. (2000) Phylogenetic species recognition and species concepts in fungi. Fungal Genetics and Biology, 31, 21–32. 11118132 10.1006/fgbi.2000.1228

[mpp13209-bib-0106] Tremblay, É.D. , Duceppe, M.O. , Bérubé, J.A. , Kimoto, T. , Lemieux, C. & Bilodeau, G.J. (2018) Screening for exotic forest pathogens to increase survey capacity using metagenomics. Phytopathology, 108, 1509–1521. 29923801 10.1094/PHYTO-02-18-0028-R

[mpp13209-bib-0107] Turgeon, B.G. (1998) Application of mating type gene technology to problems in fungal biology. Annual Review of Phytopathology, 36, 115–137. 10.1146/annurev.phyto.36.1.115 15012495

[mpp13209-bib-0108] Turgeon, B.G. & Yoder, O.C. (2000) Proposed nomenclature for mating type genes of filamentous ascomycetes. Fungal Genetics and Biology, 31, 1–5. 11118130 10.1006/fgbi.2000.1227

[mpp13209-bib-0109] Vaz, A.B. , Fonseca, P.L. , Leite, L.R. , Badotti, F. , Salim, A.C. , Araujo, F.M. et al. (2017) Using next‐generation sequencing (NGS) to uncover diversity of wood‐decaying fungi in neotropical Atlantic forests. Phytotaxa, 295, 1–21.

[mpp13209-bib-0110] Villani, A. , Logrieco, A.F. , Moretti, A. , Susca, A. , Brown, D.W. , Proctor, R.H. et al. (2015) *Fusarium incarnatum‐equiseti* species complex from cereals: phylogeny and variability of trichothecene biosynthetic gene cluster. Journal of Plant Pathology, 97, S22.

[mpp13209-bib-0111] Vitale, A. , Crous, P.W. , Lombard, L. & Polizzi, G. (2013) *Calonectria* diseases on ornamental plants in Europe and the Mediterranean basin: an overview. Journal of Plant Pathology, 95, 463–476.

[mpp13209-bib-0112] Wang, Q.C. & Chen, S.F. (2020) *Calonectria pentaseptata* causes severe leaf disease of cultivated *Eucalyptus* on the Leizhou Peninsula of Southern China. Plant Disease, 104, 493–509. 31790643 10.1094/PDIS-05-19-1009-RE

[mpp13209-bib-0113] Wang, Q.C. , Liu, Q.L. & Chen, S.F. (2019) Novel species of *Calonectria* isolated from soil near *Eucalyptus* plantations in southern China. Mycologia, 111, 1028–1040. 31634057 10.1080/00275514.2019.1666597

[mpp13209-bib-0114] West, J.S. (2014) Plant pathogen dispersal. eLs, 10.1002/9780470015902.a0021272

[mpp13209-bib-0115] Wilson, A.M. , Wilken, P.M. , van der Nest, M.A. , Steenkamp, E.T. , Wingfield, M.J. & Wingfield, B.D. (2015) Homothallism: an umbrella term for describing diverse sexual behaviours. IMA Fungus, 6, 207–214. 26203424 10.5598/imafungus.2015.06.01.13 PMC4500084

[mpp13209-bib-0116] Wilson, A.M. , Wilken, P.M. , van der Nest, M.A. , Wingfield, M.J. & Wingfield, B.D. (2019) It’s all in the genes: the regulatory pathways of sexual reproduction in filamentous ascomycetes. Genes, 10, 330. 10.3390/genes10050330 PMC6562746 31052334

[mpp13209-bib-0117] Wilson, A.M. , Wilken, P.M. , Wingfield, M.J. & Wingfield, B.D. (2021) Genetic networks that govern sexual reproduction in the Pezizomycotina. Microbiology and Molecular Biology Reviews, 85, e00020‐21. 10.1128/MMBR.00020-21 PMC8485983 34585983

[mpp13209-bib-0118] Wingfield, M.J. , De beer, Z.W. , Slippers, B. , Wingfield, B.D. , Groenewald, J.Z. , Lombard, L. et al. (2012) One fungus, one name promotes progressive plant pathology. Molecular Plant Pathology, 13, 604–613. 22146077 10.1111/j.1364-3703.2011.00768.x PMC6638803

[mpp13209-bib-0119] Wingfield, M.J. , Slippers, B. , Roux, J. & Wingfield, B.D. (2001) Worldwide movement of exotic forest fungi, especially in the tropics and the southern hemisphere: this article examines the impact of fungal pathogens introduced in plantation forestry. BioScience, 51, 134–140.

[mpp13209-bib-0120] Wright, L.P. , Davis, A.J. , Wingfield, B.D. , Crous, P.W. , Brenneman, T. & Wingfield, M.J. (2010) Population structure of *Cylindrocladium parasiticum* infecting peanuts (*Arachis hypogaea*) in Georgia, USA. European Journal of Plant Pathology, 127, 199–206.

[mpp13209-bib-0121] Wright, L.P. , Wingfield, B.D. , Crous, P.W. , Brenneman, T. & Wingfield, M.J. (2006) Isolation and characterization of microsatellite loci in *Cylindrocladium parasiticum* . Molecular Ecology Notes, 6, 110–112.

[mpp13209-bib-0122] Wright, L.P. , Wingfield, B.D. , Crous, P.W. & Wingfield, M.J. (2007) Isolation and characterization of microsatellite loci in *Cylindrocladium pauciramosum* . Molecular Ecology Notes, 7, 343–345.

[mpp13209-bib-0123] Wu, W.X. & Chen, S.F. (2021) Species diversity, mating strategy, and pathogenicity of *Calonectria* species from diseased leaves and soils in the *Eucalyptus* plantation in southern China. Journal of Fungi, 7, 73. 33498546 10.3390/jof7020073 PMC7909555

[mpp13209-bib-0124] Ye, X. , Liu, H. , Jin, Y. , Guo, M. , Huang, A. , Chen, Q. et al. (2017) Transcriptomic analysis of *Calonectria pseudoreteaudii* during various stages of *Eucalyptus* infection. PLoS One, 12, e0169598. 28072879 10.1371/journal.pone.0169598 PMC5224884

[mpp13209-bib-0125] Ye, X. , Zhong, Z. , Liu, H. , Lin, L. , Guo, M. , Guo, W. et al. (2018) Whole genome and transcriptome analysis reveal adaptive strategies and pathogenesis of *Calonectria pseudoreteaudii* to *Eucalyptus* . BMC Genomics, 19, 358. 29747580 10.1186/s12864-018-4739-1 PMC5946483

[mpp13209-bib-0126] Yoder, O.C. , Valent, B. & Chumley, F. (1986) Genetic nomenclature and practice for plant pathogenic fungi. Phytopathology, 76, 383–385.

